# Commentary: Oncoplastic Breast Reconstruction Complications and Patient-Reported Outcomes

**DOI:** 10.1177/22925503261425233

**Published:** 2026-02-27

**Authors:** Charles Arcand, Tyler Safran, Joshua Vorstenbosch

**Affiliations:** 1 Division of Plastic and Reconstructive Surgery, 54473 McGill University, Montreal, Canada

Oncoplastic breast reconstruction emerged as an alternative to breast conservation surgery with the purpose of improving postoperative aesthetic outcomes, all while preserving a high level of oncologic safety. In their study, Mysuria et al^1^ present their institutional experience with oncoplastic breast reconstruction over more than a decade, contributing valuable data about safety and patient satisfaction.

This report of 81 consecutive patients assesses complication rates and evaluates patient-reported outcomes using the validated BREAST-Q 2.0.^2^ Their findings highlight a low complication rate, with no major complication requiring an unexpected return to the operating room. Most importantly, no complication delayed the oncologic treatment of the patients with either chemotherapy or radiotherapy, reinforcing the safety of their approach. Additionally, the high level of satisfaction across multiple BREAST-Q domains further supports the role of oncoplastic breast reconstruction in optimizing patient satisfaction while maintaining oncologic safety.

While the authors do well to highlight the benefits of oncoplastic breast reconstruction, some aspects of the study merit further discussion. First, while the low complication rate is encouraging, the relatively small sample and their reliance on univariate analysis limit the ability to draw definite conclusions regarding the independent risk factors, such as the radiation status or the tumor size. Second, the low completion rate of the BREAST-Q introduces a potential sampling bias, and the lack of preoperative or longitudinal postoperative measurements limits assessment of change over time. In future studies, prospective collection of serial patient-reported outcomes could strengthen future analysis, particularly in the case of long-term radiation-induced changes.

Finally, we applaud the efforts of these two fellowship-trained surgeon groups in spearheading the research efforts in oncoplastic breast surgery and hope that it will inspire feasibility in broader settings beyond major academic centers.

Overall, Mysuria et al^1^ provide an important contribution to a growing body of evidence supporting oncoplastic breast reconstruction. The conclusions drawn echo the works of DiPasquale et al^3^ and Gardfjell et al,^4^ who found a similar rate of breast satisfaction. Further studies gathering serial patient-reported outcomes and complication rates will assist cancer patients in choosing the most appropriate approach to their breast reconstruction. Lastly, these favorable outcomes will enable breast cancer patients to enhance their informed decision-making process and could drive further collaboration between oncologic breast surgeons and plastic surgeons to increase access to oncoplastic breast surgery.
